# Unequal Labour Market Impacts of COVID-19 in Sweden — But Not Between Women and Men

**DOI:** 10.1007/s10272-021-0996-3

**Published:** 2021-10-05

**Authors:** Pamela Campa, Jesper Roine, Svante Strömberg

**Affiliations:** Department of Economics, Stockholm School of Economics, Sveavägen 65, Box 6501, SE-113 83 Stockholm, Sweden

## Abstract

So, when asked if men and women in the Swedish labour market have been differently affected by the COVID-19 pandemic, our overall conclusion would have to be “no”.

As the COVID-19 pandemic hit the world economy in the beginning of 2020, concerns were soon raised about potentially increasing inequality in many dimensions.[1] With respect to gender differences, an early study by Alon et al. (2020) pointed to a number of reasons why the pandemic recession, unlike traditional ones, may become more severe for women. Subsequent studies have found mixed evidence. On the one hand, based on survey data from the UK and the US (Adams-Prassl et al., 2020), and based on administrative data from the US (Cajner et al., 2020), negative labour market outcomes have indeed been worse for women. On the other hand, Adams-Prassl et al. (2020) found no evidence of unequal impacts by gender in Germany, nor was it the case in Italy according to Casarico and Lattanzio (2020). At present, it does ap-

1 See, for instance, Goldin and Muggah (2020) and Stiglitz (2020).

pear that the effects, on average, disadvantage women, however, labour market outcomes by gender vary across countries (see e.g. Hupkau and Petrongolo, 2020; Bluedorn et al., 2021; and Alon et al., 2021).

From a policy point of view, it is interesting to try to distinguish between the importance of pre-existing differences between men and women in the labour market and specific policy responses to the pandemic. Is the more important factor gender equality in the country before the pandemic or the government's response to the pandemic? In both respects, the case of Sweden stands out. Sweden is of course well known for its overall gender equality: Labour market participation is high for both men and women, and the gap is relatively small; gender wage gaps are relatively low and shrinking; conditions allowing people to combine career and family are good, and possibilities to do so are formally gender neutral. At the same time, a number of differences remain: Horizontal labour market segregation between men and women remains important; in the vertical dimension, there are still very few women in top paying positions; and in practice, childcare and household responsibilities remain relatively unequally shared. With respect to the pandemic response, Sweden has famously (or infamously) also been different. Even if the "business-as-usual" portrayal in some media has not quite been the case, it remains a fact that Sweden has not imposed as many restrictions as most other countries. In particular, the government never imposed any stay-at-home orders, which meant that jobs were not distinguished as being either "essential" or "non-essential". The reliance on recommendations and individual responsibility led to some sectors being severely impacted, but never entirely shut down. Also, very importantly, daycare facilities and schools below secondary level (around age 15) never closed in Sweden.[2]

So what has been the impact of COVID-19 on men's and women's respective labour market outcomes in Sweden?

2 See Ellingsen and Roine (2020) for an account of Sweden's response to COVID-19 in comparison to the other Nordic countries.

Figure 1
**Unemployment rate for men and women and the change in the employment gap**

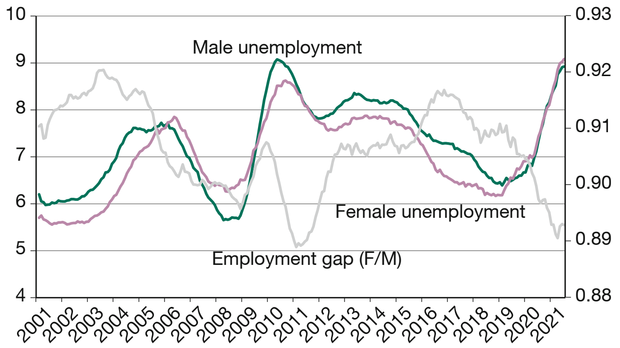

Note: The unemployment rate for men and women is in percent relative to population (seasonally adjusted, LHS) and the change in the employment gap is the ratio of female/male employment rate (RHS).

Source: Labor Force Survey, Statistics Sweden.

This article provides an overview of some results, drawing heavily on our recent work on unemployment inequalities in Sweden during the first phase of the pandemic (Campa et al., 2021), as well as some additional insights.

## Labour market impacts of COVID-19 in Sweden

As in most other countries, the Swedish labour market was heavily hit by the COVID-19 pandemic. In 2020, the highest unemployment rate was recorded at 9.2%, higher than the 8.9% peak during the 2008 financial crisis. With respect to differences between men and women, Figure 1 shows men's unemployment exhibiting larger volatility in the financial crises (2008-2011), but the increase in the recent crisis has been almost equal between men and women (even slightly larger for women) since March 2020. At the same time, women's employment rate has gone down a little more than men's, causing the employment gap to decrease.[3]

Another important aspect when looking at the labour market impact of the pandemic in Sweden was the extensive use of state-subsidised furlough schemes. The Swedish government introduced furloughs in mid-March 2020, at a cost of approximately US $4.2 billion by the end of that year (Regeringskansliet, 2021).[4]

3 A drop in this measure is used in Bluedorn et al. (2021) as the definition of a country experiencing a she-cession in the pandemic where Sweden is indeed defined as having experienced this.

4 https://www.regeringen.se/regeringens-politik/regeringens-arbete-med-coronapandemin/foretag/om-forslaget-korttidspermittering/

Figure 2
**Number of furloughed and unemployed in Sweden, 2020**

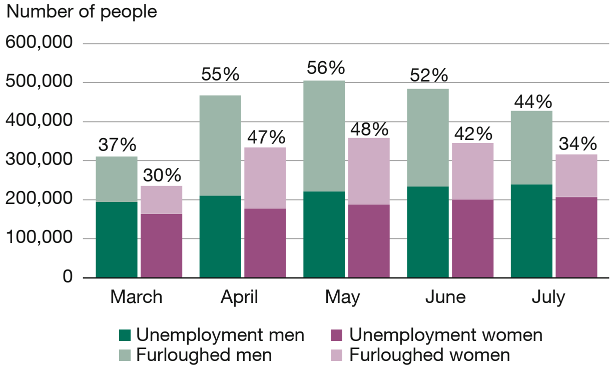

Note: The percentages indicate the share of furloughed workers relative to the total unemployment pool (furloughed and unemployed).

Source: Swedish Public Employment Service and Swedish Agency for Economic and Regional Growth.

Figure 3
**Employed persons in Sweden by age, 2020**

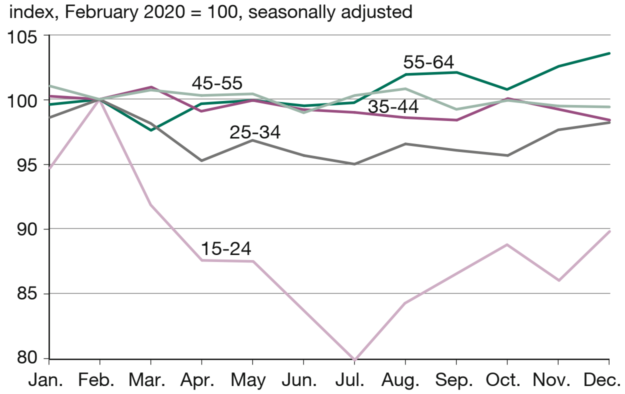

Source: Labor Force Survey, Statistics Sweden.

Figure 2 illustrates the number of unemployed in relation to furloughed workers during the first five months of the pandemic. Between March and April, the number of people entering furlough increased drastically by 225,000 individuals.[5] The total number makes up a considerable share of the total pool of individuals “currently not working” (sum of furloughed and formally unemployed). In terms of differences between men and women, aggregate unemployment was relatively similar when considering that women have slightly lower labour force participation than men (Statistics Sweden, 2021a). But when furloughs were added, men were more affected. In May 2020, the shares of furloughed workers in relation to the labour force was about 9.7% and 6.5% for men and women, respectively, and this gap persists throughout the period depicted in the figure. This is likely because the sector with the highest number of furloughed workers is manufacturing, a predominantly male sector with only 24% of female employees.

Looking at other dimensions, age (see Figure 3) and being foreign born stand out. The unemployment rate increased most rapidly, by 2.2 percentage points between February and June 2020. In this period, the corresponding increase for people aged 15-24 was 7.2 percentage points and for those born outside of Sweden 3.7 percentage points (Statistics Sweden, 2021b).

5 In the period after that, as shown in Figure 2, when the second and third waves hit, the number of furloughed has not peaked at all in the same way as in the first phase, but rather stayed relatively stable.

Individuals working in the service sector, particularly those in the transportation and hotel/restaurant industries, were hit the hardest by the restrictions and recommendations put in place to limit the spread of the pandemic. Workers who had temporary contracts were also more affected. Among those with temporary contracts, the number of employed decreased by 12% in the first half of 2020 (Statistics Sweden, 2021b). People with permanent contracts were not as impacted in terms of employment and by December had almost recovered to the levels they were at prior to the pandemic. Currently, the biggest worry is the increase in long-term unemployment. A quarter of the total number of people who registered as unemployed between March and May 2020 were still jobless one year later, thus qualifying as long-term unemployed.

## Changes in unemployment risk in the pandemic

A closer look at these dimensions and their interactions in Campa et al. (2021) reveals a number of more precise results. We use monthly data with population-wide coverage from the registry of unemployed for the periods February to July in 2019 and in 2020, collected and kept by the Swedish Public Employment Service. The data is on the four-digit sector level (amounting to 400 occupations) and disaggregated by gender, age and foreign-born status. For each group, we construct a measure we call “unemployment risk”, an occupation-level monthly measure defined as the ratio of registered unemployed in 2019 and 2020 divided by the average group level employment in the previous two years. To study the impact of the pandemic, we then look at the change in unemployment risk between one month before the start of the pandemic (February 2020) and five months after.[6] In addition, we complement this data with information on the average wage by occupational group and gender as reported by *Medlingsinstitutet* and publicly available at Statistics Sweden.

Our results on the impact of the pandemic on unemployment risk confirm some of the above observations. First, the risk of becoming unemployed increased significantly for young workers. One explanation could be that young people are concentrated in occupations that are heavily impacted by government response policies (e.g. bartenders, waiters or café personnel). However, when adding occupation fixed effects, we find that this only explains part of the negative relationship between age and change in unemployment risk. Even within occupations, young people have been more likely to enter unemployment relative to their older colleagues. This, in turn, can be explained by a Swedish labour market institution, according to which the order of downsizing is determined by seniority; the last one hired is the first one fired, typically the youngest age groups. Second, we confirm that the pre-pandemic risk of becoming unemployed varies significantly across foreign-born status: Relative to Sweden-born workers, the unemployment risk for those born in other EU countries was six percentage points higher, and an astonishing 25 percentage points higher for those born outside of the EU. Importantly, this foreign-born gap is not explained by occupational sorting and has also widened during the pandemic. A third general result, not shown in the above, is that there is a clear wage gradient in the change in unemployment risk due to the pandemic. As shown in Figure 4, unemployment risk is inversely related to wage level, and this relation has become stronger in the pandemic.

As we know that women are overrepresented in lower paying occupations, this could indicate that women were more affected by the pandemic. However, the one dimension where we find very small differences is gender. Overall, there is no significant difference between men and women in the risk of losing one's job due to the pandemic. If anything, when we study intersectoral differences, the change in unemployment risk has increased slightly more for men than women. Interestingly, the female advantage relative to men is weakened when accounting for age, suggesting that differences in the gender composition across occupation-by-age groups partly explains the (small but) unequal gender impact of the pandemic. In fact, we see in our data that the largest predominantly

Figure 4
**Unemployment risk across wages in 2019 and 2020**

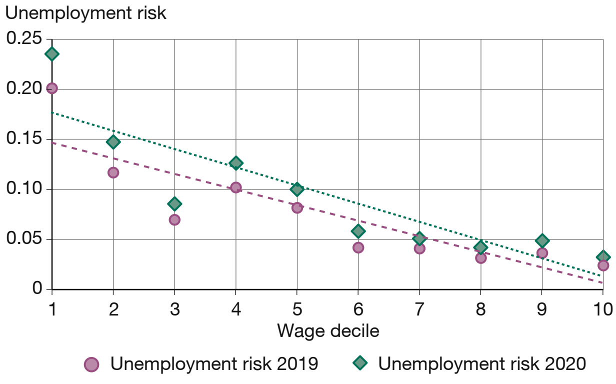

Source: Authors' own calculations based on data from the Swedish Public Employment Service and Statistics Sweden.

female and male occupations,[7] respectively, are more gender balanced in the younger age groups. Occupations that traditionally have been dominated by women (men) have experienced an inflow of young male (female) workers over time. This demographic development has evolved faster in occupations dominated by women than those dominated by men. Then, since men tend to be younger than women, and employers follow the "first in – last out" policy, we should see a disproportionate number of men losing their jobs primarily due to their age in these occupations. The same applies to male-dominated occupations but to a lesser extent. The initial gender difference is also accentuated when we control for foreign-born status, confirming that female workers tend to differ from male workers on some characteristics that are important determinants of the unemployment impact of the pandemic, especially within occupations.

## No differences between men and women in Sweden?

Taken together, the above results show that the COVID-19 pandemic indeed has had some very unequal effects on different groups in the labour market, but not between men and women. The employment rate did drop a little more for women, but unemployment rates, and increases in unemployment rates have been relatively equal, and when adding furloughed individuals, men were more affected. When looking closer at changes in unemployment risk, the differences between men and women are again

6 See Campa et al. (2021) for details.

7 We define an occupation to be predominantly female (male) if the number of female workers is above 60% (below 40%).

Figure 5
**Unemployment risk and gender composition of sectors**

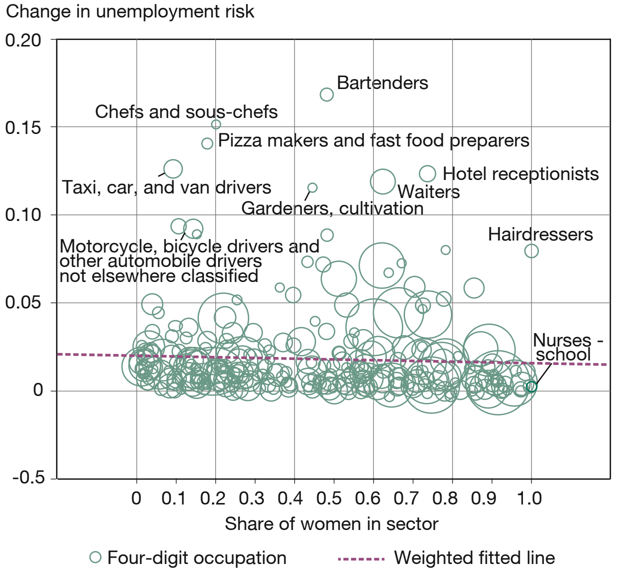

Note: The unit of observation is four-digit occupation. Marker size and fitted line are weighted by number of employed. We exclude occupations that contain below 2500 employees.

Source: Authors' own calculations based on data from the Swedish Public Employment Service and Statistics Sweden.

small. So what explains the lack of gender differences in the labour market impact of COVID-19 in Sweden?

Figure 5 gives a first hint. One of the initially identified reasons for why women may be more affected in the pandemic recession was that they work in sectors more affected by social distancing measures (see e.g. Alon et al., 2020). In Sweden this is not the case, at least not ex post. Figure 5 shows the increased unemployment risk in the pandemic across sectors by the share of women in the sector. The weighted fitted line is virtually straight as women-dominated sectors did not experience a larger increase in unemployment risk. This result is based on a mix of pre-pandemic factors and policy choices. Some of the sectors that saw an increased demand for labour, such as the health sector in particular, were in many cases already dominated by women before the pandemic. Assistant nurses, for example, where the share of women is 91%, experienced well below-average unemployment risk. The same is true for many other occupations in health care that are also dominated by women. In other occupations, the policy responses matter. The fact that there were no primary school closures meant that unemployment risk in this occupation was also low, to the benefit of primary school teachers – of whom 75% are women.[8]

Another point that has been raised as a potential cause for worse labour market outcomes for women in the pandemic has been the so-called telecommutability of different occupations. Alon et al. (2020) find that, in the US, men are more likely than women to be in occupations that are telecommutable, suggesting that women will have a harder time adapting to stay-at-home and social-distancing orders. Even though the Swedish government did not adopt such policies formally, many workplaces did follow recommendations to work from home and therefore this could still be a factor. However, looking at telecommutability and the share of women across occupations in Sweden, we do not find this to be to the disadvantage of women.[9]

In Figure 6, we plot the relationship between the extent to which an occupation can be done from home and the share of women across two-digit occupations, weighted by size. The slope of the line indicates that male-dominated occupations are slightly less suited to be performed outside of the workplace.[10] However, when regressing the share of female workers in sector and telecommutability against change in unemployment risk, we find no significant relationship (even when only including the share of women as the explanatory variable). This is likely due to the small sample size as our telecommutability measurement only varies at the two-digit sector level. In any case, if our data indicates anything, it is that men should be more affected by the pandemic since they tend to work in sectors with a lower share of telecommutable jobs, in contrast to the situation in the US.

8 Compared to the sector average of 0.0274, the change in unemployment risk due to the pandemic was 0.0047 for assistant nurses, home care and homes for the elderly; 0.0034 for primary school teachers.

9 To approximate people's ability to work remotely, we adopt the classification developed by Dingel and Neiman (2020). This variable is an approximate measure of the share of jobs that can be done from home by occupational groups, in accordance with the International Standard Classification of Occupation (ISCO). Since we use a different occupational classification we add two-digit SSYK codes to the associated two-ISCO categories using a translation key. Then, using data on the number of employed by ISCO-category (available at ILOSTAT), we calculate the arithmetic mean of the share of jobs that can be done from home by SSYK occupation, with the ISCO employment counter as weights.

10 The top three two-digit occupations with the highest share of male (female) workers include "Electrical and electronic trades workers", "Building and construction workers" and "Metal, machinery and related trades workers" ("Office clerks", "Personal care workers" and "Occupations requiring advanced academic competence in health care").

Figure 6

**Telecommutability and gender composition of sector**

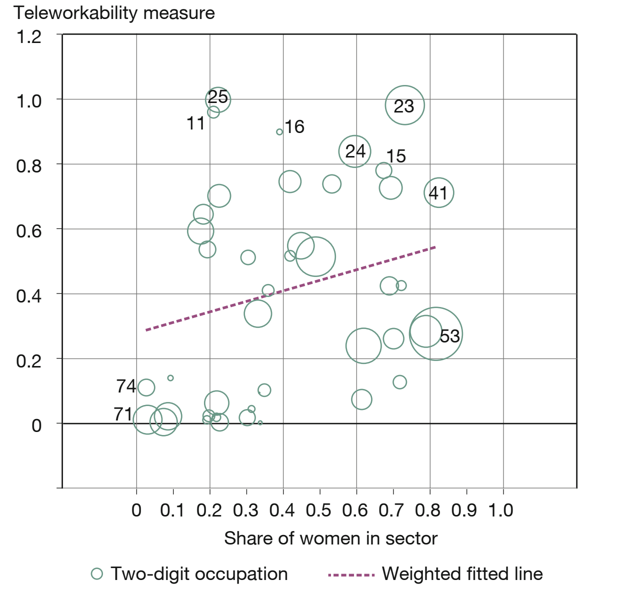

25 - Occupations requiring advanced academic skills in information and communications technology
23 - Occupations requiring advanced academic competence in education
11 - Legislators, chief executives and senior government officials
16 - Financial and insurance services branch managers
24 - Occupations requiring advanced academic competence in finance and management
15 - Health and other services managers
41 - Office clerks
53 - Personal care workers
74 - Electrical and electronic trade workers
71 - Building und construction workers

Note: The unit of observation is two-digit occupation. Marker size and fitted line are weighted by number of employed.

Source: Statistics Sweden.

Yet another point which has been raised as a key reason for why a pandemic may have more of an impact on women's labour market outcomes is that women often take on more of childcare and household duties. As concisely phrased by Hupkau and Petrongolo (2020): "During lockdowns, virtually none of the typical components of home production could be outsourced to the market, and the closure of schools and nurseries meant that all education and childcare services were added to pre-existing home production needs". This was typically to the disadvantage of women. In the case of Sweden, however, schools and nurseries were never closed, causing a double benefit to women's labour market outcome. As previously noted, these activities employ a larger share of women who therefore faced less unemployment risk; but it also meant that women, who (also in Sweden) are more likely to be at home with children who cannot go to school or daycare, could continue working.

## Conclusion

So, when asked if men and women in the Swedish labour market have been differently affected by the COVID-19 pandemic, our overall conclusion would have to be "no". There are several other factors that stand out as important, in particular age and being foreign born. Of course, the impact has varied greatly across different sectors and occupations, but in general the differences between men and women have been small. There are several, sometimes overlapping and interacting, reasons for this. First, even if the Swedish labour market is horizontally segregated between men and women, it does not seem to be in a way that disadvantages women in the pandemic, nor in ways that would make working from home more difficult for women on average. Second, a number of policy choices, in particular keeping schools and day cares open have also worked to the advantage of women in terms of employment outcomes.
